# Trend in alcohol use in Australia over 13 years: has there been a trend reversal?

**DOI:** 10.1186/s12889-016-3732-3

**Published:** 2016-10-10

**Authors:** Gary C.K. Chan, Janni K. Leung, Catherine Quinn, Jason P. Connor, Leanne Hides, Matthew J. Gullo, Rosa Alati, Megan Weier, Adrian B. Kelly, Wayne D. Hall

**Affiliations:** 1 Centre for Youth Substance Abuse Research, The University of Queensland, Brisbane, QLD 4072 Australia; 2 Policy and Epidemiology Group, Queensland Centre for Mental Health Research, Brisbane, Australia; 3 School of Public Health, The University of Queensland, Brisbane, Australia; 4 Centre for Youth Substance Abuse Research, Queensland University of Technology, Brisbane, Australia; 5 Discipline of Psychiatry, School of Medicine, The University of Queensland, Brisbane, Australia

**Keywords:** Alcohol, Drinking, Epidemiology, Trend, prevalence

## Abstract

**Background:**

Skog’s collectivity theory of alcohol consumption predicted that changes in alcohol consumption would synchronize across all types of drinkers in a population. The aim of this paper is examine this theory in the Australian context. We examined whether there was a collective change in alcohol use in Australia from 2001 to 2013, estimated alcohol consumption in non-high risk and high risk drinkers, and examined the trends in alcohol treatment episodes.

**Methods:**

Data from the 2001–2013 National Drug Strategy Household Surveys (*N =* 127,916) was used to estimate the prevalence and alcohol consumption of abstainers, high risk drinkers and frequent heavy episodic drinkers. Closed treatment episodes recorded in the Alcohol and Other Drug Treatment Services National Minimum Dataset (*N =* 608,367) from 2001 to 2013 were used to examine the trends of closed alcohol treatment episodes.

**Results:**

The prevalence of non-drinkers (abstainers) decreased to the lowest level in 2004 (15.3 %) and rebounded steadily thereafter (20.4 % in 2013; *p* < .001). Correspondingly, the per capita consumption of high risk drinkers (2 standard drinks or more on average per day) increased from 20.7 L in 2001 to peak in 2010 (21.5 L; *p* = .020). Non-high risk drinkers’ consumption peaked in 2004 (2.9 L) and decreased to 2.8 L in 2013 (*p* < .05). There were decreases in alcohol treatment episodes across nearly all birth cohorts in recent years.

**Conclusion:**

These findings are partially consistent with and support Skog’s collectivity theory. There has been a turnaround in alcohol consumption after a decade-long uptrend, as evident in the collective decreases in alcohol consumption among nearly all types of drinkers. There was also a turnaround in rate of treatment seeking, which peaked at 2007 and then decreased steadily. The timing of this turnaround differs with level of drinking, with non-high risk drinkers reaching its peak consumption in 2004 and high risk drinkers reaching its peak consumption in 2010.

## Background

Alcohol use is a cause of 60 different diseases [1] and contributes to over three million deaths worldwide each year [2]. In Australia, it is a leading cause of preventable deaths and hospitalizations, contributing to 4.28 % of total Disability Adjusted Life Years (DALYs) and 4.27 % of total deaths in 2013 [3]. Alcohol use is also a significant financial burden. In 2010, the total cost of alcohol-related problems in Australia was over $14 billion [4]. The worldwide per capita alcohol consumption was 6.2 L in 2010, for persons above 15 years. It was 11.8 L in the United Kingdom, 10.2 L in Canada, 9.2 L in the USA, and 12.2 L in Australia [2].

Monitoring and reducing per capita consumption has been a central focus of many international alcohol policies and prevention efforts because alcohol related harm in the population is strongly associated with population alcohol consumption [5]. It has also been suggested that the per capita consumption reflects a countries’ drinking culture, and that changes in alcohol consumption would synchronize across all types of drinkers in a population, from lighter to heavier drinkers [6]. Skog’s theory of collectivity of alcohol use [6] provides a social explanation for this collective change in alcohol use across the whole population. Drinking is a social phenomenon, and drinkers learn societal drinking norms through interaction with others. Drinkers are strongly influenced by the alcohol use behaviour of their peers while at the same time influence the drinking behavior of others. These mutual influences between drinkers underlie the collective changes of alcohol use in a population. A prediction of this theory is that changes in per capita consumption should be accompanied by changes in consumption in all types of drinkers and alcohol-related harms. There is reasonable empirical evidence for this prediction, with lower per capita consumption predicting a lower prevalence of heavy drinkers, lower consumption across the distribution of consumption, and lower levels of alcohol related harm in the population [5, 7, 8].

Recent studies in Australia have reported diverging trends in alcohol use and alcohol related harms. Among young Australians, for example, the abstinence rate has increased in the last few years but so too has alcohol-related harm and injury [9, 10]. Across the whole Australian population, the number of alcohol-related hospitalizations increased by over 50 % between 2001 and 2010 during a period in which per capita consumption increased by only 3 % [11] and the abstinence rate among Australian adolescents increased by 50 % [9]. A similarly diverging trend has been reported in other high income countries such as Sweden [12] and the UK [13].

Polarization of drinking patterns has been proposed as an explanation of the diverging trend in mean level of consumption in the population and alcohol related harm [13]. This hypothesis is that the consumption has decreased most among light drinkers, more of whom now abstain, while heavy drinkers have engage in riskier drinking.

The aim of this paper is to examine evidence on the collectivity and polarization hypothesis in Australian drinkers. This paper presents population data on changes in alcohol use and treatment seeking, and examines their trends across different types of drinkers over a 13-year period. Two primary data sources were used-the National Drug Strategy Household Surveys (NDSHS) and Alcohol and Other Drug Treatment Services National Minimum Dataset (AODTS-NMDS). The NDSHSs are the largest nationally representative data on alcohol use in Australia. The AODTS-NMDS recorded treatment episodes in all publicly funded government and non-government agencies that provide alcohol and other drug treatment services. National surveys may underestimate alcohol use due to under-reporting and non-response bias [14]. National treatment data provide information on Australians with more severe alcohol-related problems. Therefore, survey and treatment data together provide a more comprehensive overview of alcohol use and harm in Australia.

## Method

### Survey data on alcohol use

#### Sample

The samples were drawn from the tri-annual consecutive NDSHSs conducted between 2001 and 2013. The 2013 survey was the most recent nationally representative data on alcohol use in Australia and these data were released in 2015. The NDSHSs were conducted in all Australian states and territories, with an overall sample size of over 23,000 at each survey. The total sample size used in this study was 127,916. The NDSHSs are designed to be representative of the Australian population aged 14 or above, and the survey was weighted to adjust for any disparity arising from its implementation, and to align the samples with the Australian population.

Households were randomly selected using a multi-stage stratified design based on statistical local areas [15] and the respondent was the household member aged 14 years or older whose birthday was next to occur in the family. Data were predominately obtained through a ‘drop and collect’ method across the five surveys (60–100 %). Self-completion questionnaires were delivered and collected to/from households. For the 2001, 2004 and 2007 surveys, data collection was augmented by face-to-face interviews and/or Computer-Assisted Telephone Interviews. The response rates are between 46 to 56 % across surveys and this was comparable to other large scale Australian and international drug and alcohol survey [16, 17]. Detailed description of the sample characteristic and procedure can be found elsewhere [18–20].

#### Measures

Four dimensions of alcohol use were assessed. First, *abstinence* was measure “Have you had an alcoholic drink of any kind in the last 12 months”. Second, *alcohol consumption* was estimated by graduated frequency measures. The response range for quantity was none/1–2 drinks/3–4 drinks/5–6 drinks/7–10 drinks/11–19 drinks and 20 or more drinks and the response range for each quantity response were everyday/5–6 days a week/3–4 days a week/1–2 days a week/2–3 days a month/about 1 day a month/less often and never. The total volume consumed by each participant was calculated by multiplying the frequency and quantity (using midpoints determined by log-normal distribution to adjust for bias arising from the skewed distribution of alcohol consumption) [21]. For the quantity measure, the upper bound of the highest category (20 or more drinks) was set to be 50 (i.e. 20–50 drinks), and the midpoint estimated using this upper bound was 27. Various upper bounds were set to examine the robustness of our analyses and the results were similar. For participants who reported more than 365 drinking occasions, their consumption was calculated from their heaviest 365 occasions. Third, *high risk drinking* was defined as consuming two standard drinks or more on average per day in accordance with the Australian National Health and Medical Council guidelines [22] and consistent with estimated lifetime risk thresholds for alcohol-related mortality [23, 24]. Fourth, *frequent heavy episodic drinking* was defined as drinking 5 or more standard drink in a day at least monthly.

### Administrative data on treatment service use

#### Sample

The number of closed treatment episodes for alcohol from 2002 to 2013 was derived from the AODTS-NMDS. This dataset contains information on treatment episodes in all publicly funded government and non-government agencies that provide specialist alcohol and/or other drug treatment service. Over 90 % of the agencies submitted data to the AODTS-NMDS. There were 608,367 alcohol treatment episodes during the study period. A treatment episode (which could be inpatient or outpatient) was considered closed when one of the following conditions applied: (1) the treatment was completed or had ceased; (2) there had been no contact between the client and treatment provider for 3 months; and (3) there was a change in the main treatment type, principal drug of concern or delivery setting. Data access was approved by the Australian Institute of Health and Welfare and use of these data was approved by The University of Queensland Human Ethics Committee.

### Data analysis

All analyses were performed using STATA 13 [25]. For the NDSHS dataset, the *svy* command was used to estimate prevalence statistics and the corresponding confidence intervals to account for the complex survey design. Differences in prevalence across years were assessed using designed based F-statistics. Differences in consumption across years were assessed using t-statistics. There were 0.9–4.5 % missing data in alcohol measures across different survey years, and pair-wise deletion was used in prevalence estimation.

For the AODTS-NMDS dataset, numbers of treatment episodes across years were plotted by birth cohorts and age groups. The total population of each birth cohorts were obtained from Australian Bureau of Statistics census data and used as the denominator for calculating the treatment rate per 100,000 people. All the statistics from AODTS-NMDS were population statistics (as opposed to sample statistics) and any differences represent actual differences in the population. All the findings were summarized in figures shown in the results section. All the actual estimates, corresponding confidence intervals and population statistics used to produce these figures were shown in Appendix.

## Results

### Alcohol use of non-high risk and high risk drinkers

**Fig. 1:**
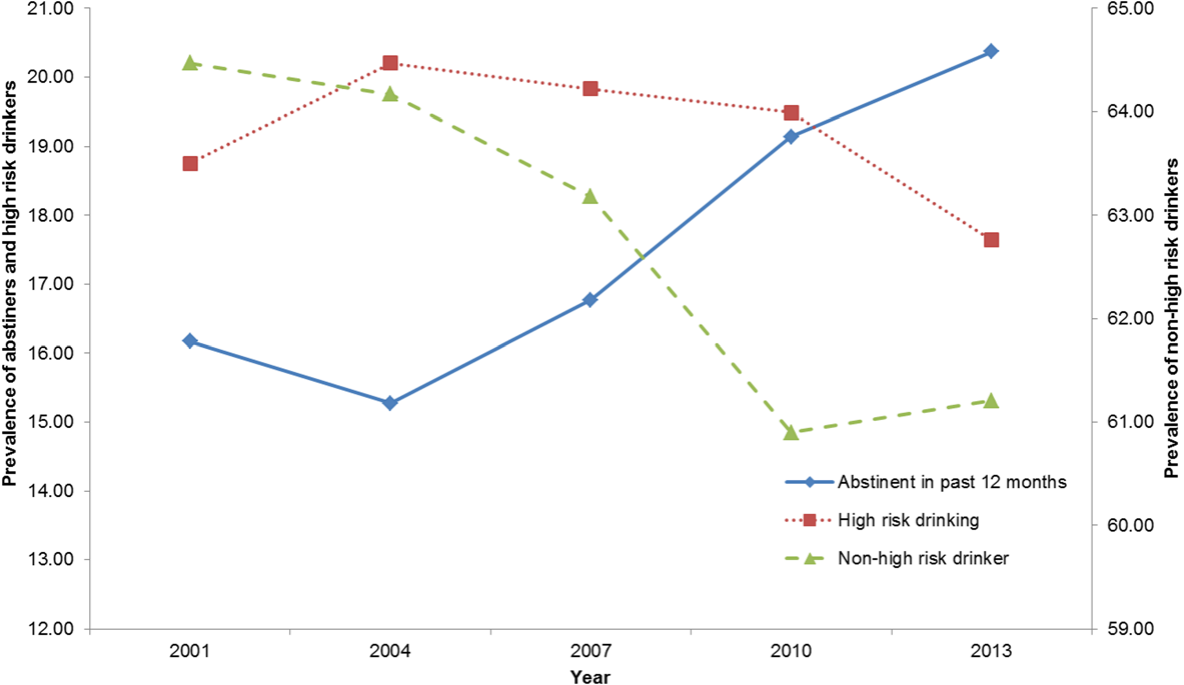
Prevalence of abstainers, high risk drinkers and non-high risk drinkers, 2001–2013

Figure 1 shows the changes in abstainers, non-high risk drinkers and high risk drinkers from 2001 and 2013. The prevalence of abstainers significantly decreased between 2001 and 2004 (*F* (1, 54 985) = 5.74, *p* = .02), from 16.2 % (95 % CI: 15.7–16.7 %) to 15.3 % (95 % CI: 14.8–15.8 %), and has increased steadily since 2004. The rate of abstinence in 2013 (20.2 %; 95 % CI: 19.6–21.0 %) was significantly higher than in 2004, *F* (1, 51 909) = 149.0, *p* < .001. The prevalence of high risk drinkers significantly increased between 2001 (18.8 %; 95 % CI: 18.2–19.3 %) and 2004 (20.2 %; 95 % CI: 19.6–20.8 %), *F* (1, 54 504) = 12.09, *p* = .001; remained fairly stable between 2004 and 2010 (19.5 %; 95 % CI: 18.9–20.1 %) with no significant changes in prevalence; and then decreased significantly from 2010 to 2013 (17.6 %; 95 % CI: 17.1–18.2 %), *F* (1, 47 999) = 18.77, *p* < .001. The prevalence of non-high risk drinkers decreased significantly from 64.5 % (95 % CI: 63.78–65.16 %) in 2001 to 60.9 % (95 % CI: 60.2–61.6 %) in 2010, *F* (1, 52 127) = 48.28, *p* < .001, and remained stable between 2010 and 2013 (61.2 %; 95 % CI: 60.4–62.0 %).

**Fig. 2:**
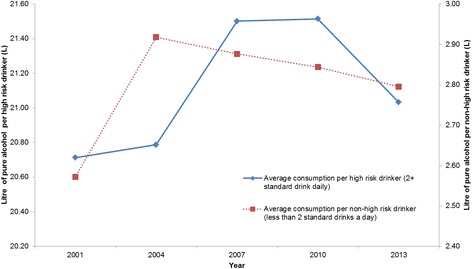
Estimated consumption of high risk drinkers and non-high risk drinkers, 2001–2013

The average consumption of non-high risk drinkers increased from 2.57 L (95 % CI: 2.53–2.62 L) a year in 2001 to 2.92 L (95 % CI: 2.87–2.94 L) in 2004, *t* = 10.39, *p* < .001, and decreased steadily to 2.80 L (95 % CI: 2.74–2.85 L) in 2013, *t* = 3.49, *p* < .001 (see Fig. 2). The average consumption of high-risk drinkers increased from 20.71 L (95 % CI: 20.22–21.20 L) in 2001 and peaked at 21.52 L (95 % CI: 21.02–22.01 L) in 2010, *t* = 2.29, *p* = .02. Although, the decrease in consumption from 2010 to 2013 (21.03 L; 95 % CI: 20.51–21.55 L) was non-statistically significant, *t* = 1.34, *p* = .181, the average consumption of high risk drinkers in 2013 was not significantly different from the average consumption in 2001, *t* = 0.88, *p* = .380, suggesting that the consumption level has returned to the 2001 level.

### Alcohol use of non-frequent and frequent heavy episodic drinkers

**Fig. 3:**
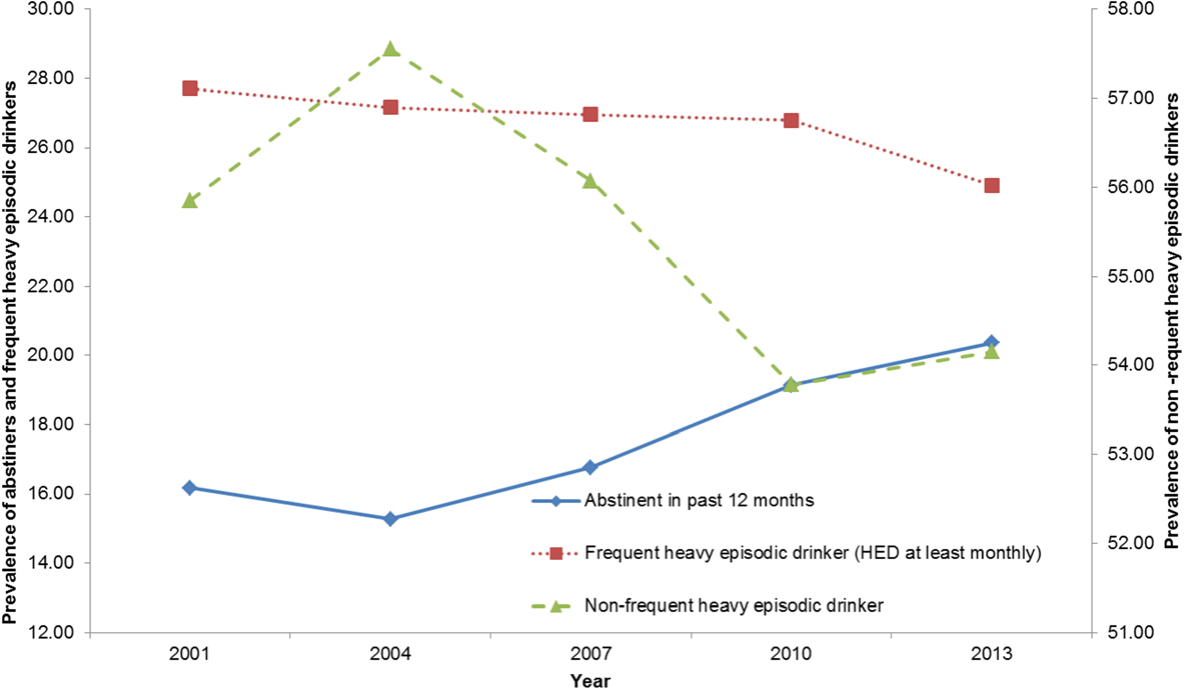
Prevalence of abstainers, frequent heavy episodic drinkers, and non-frequent heavy episodic drinkers, 2001–2013

The changes in prevalence of frequent heavy episodic drinkers and non-frequent heavy episodic drinkers are shown in Fig. 3. The prevalence of frequent heavy episodic drinkers decreased steadily from 27.7 % (95 % CI: 27.1–28.4 %) in 2001 to 24.91 % (95 % CI: 24.2–25.6 %) 2013, *F* (1, 49001) = 32.72, *p* < .001. The prevalence of non-frequent heavy episodic drinkers increased significantly from 55.9 % (95 % CI: 55.1–56.6 %) in 2001 to 57.6 % (95 % CI: 56.7–58.3 %) in 2004, *F* (1, 54565) = 11.11, *p* < .001, and decreased to 54.2 % (95 % CI: 53.4–54.9 %) in 2013, *F* (1, 51293) = 40.78, *p* < .001.

**Fig. 4:**
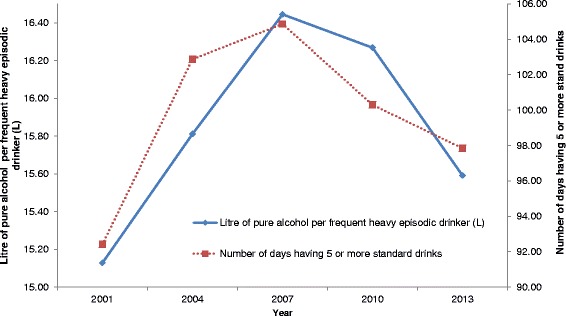
Estimated consumption and number of days engaged in heavy episodic drinking among frequent heavy episodic drinkers, 2001–2013

Figure 4 shows the changes in average yearly consumption for frequent heavy episodic drinkers and the number of days of heavy episodic drinking. Both the number of days of heavy drinking and overall yearly consumptions increased significantly from 2001, peaked at 2007 (*p* < .001) and dropped significantly in 2013 (*p* < .05). The number of days of heavy episodic drinking was still significantly higher in 2013 (97.8 days; 95 % CI: 94.4–101.3 days) than in 2001 (92.4 days; 95 % CI: 89.4–95.5 days) despite its recent drop, *t* = 2.31, *p* = .021, but the yearly consumption in 2013 (15.59 L; 95 % CI: 15.14–16.04 L) was no longer significantly higher than in 2001 (15.13 L; 95 % CI: 14.72–15.53 L), *t* = 1.49, *p* = .135.

### Trends in treatment seeking

**Fig. 5:**
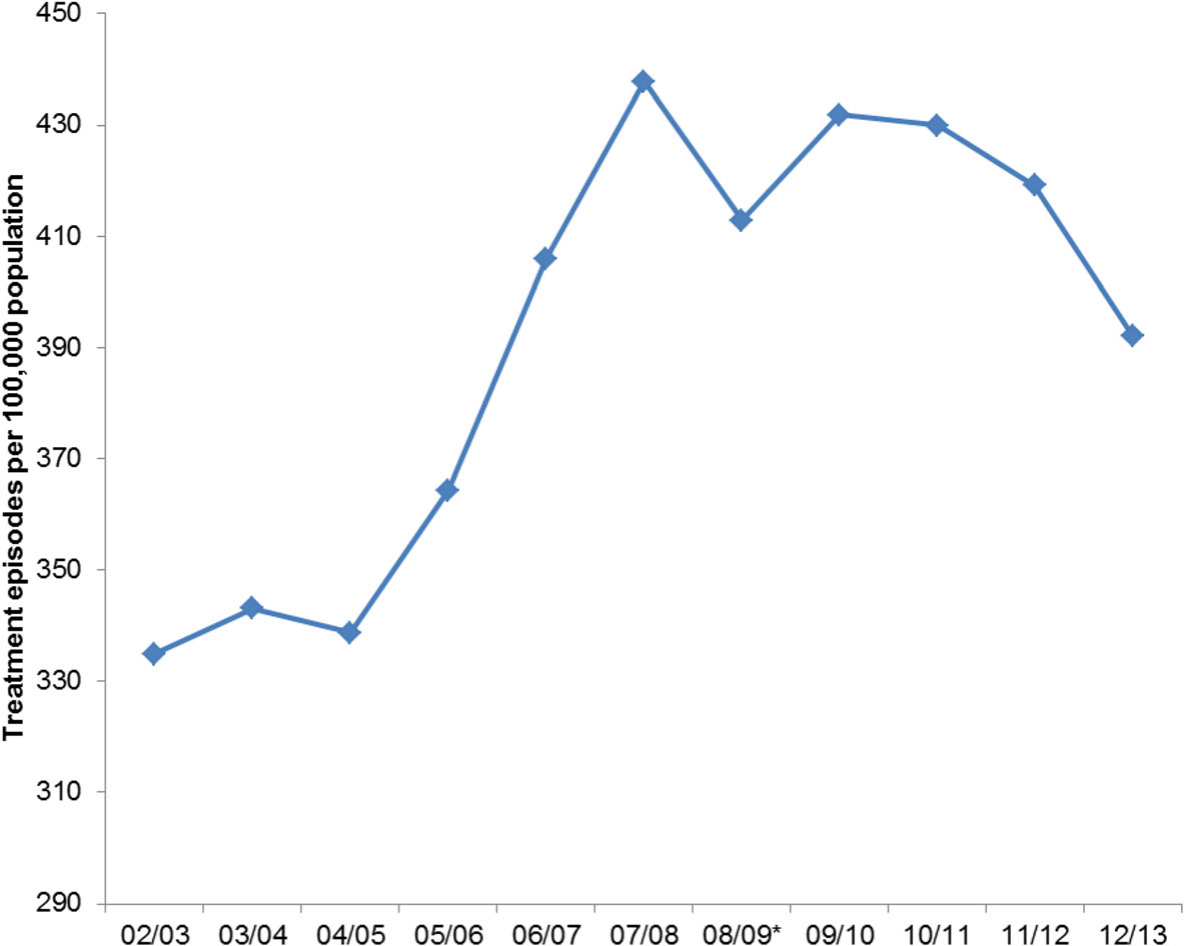
Number of treatment episodes per 100,000 population. *Numbers have dropped as New South Wales data was incomplete in 2008/2009

**Fig. 6:**
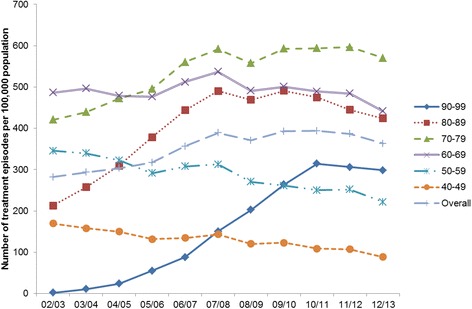
Number of treatment episodes per 100,000 population by birth cohort. *Numbers have dropped as New South Wales data was incomplete in 2008/2009

**Fig. 7:**
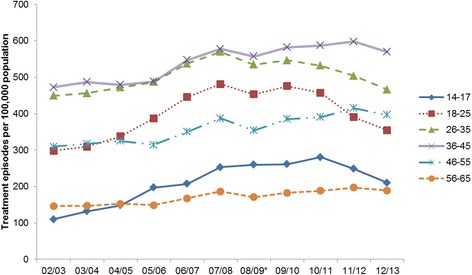
Number of treatment episodes per 100,000 population by age group. *Numbers have dropped as New South Wales data was incomplete in 2008/2009

The overall treatment rate increased from 335 per 100,000 population in 2002 to 438 per 100,000 population in 2007, and then decreased to 392 per 100,000 (Fig. 5). The treatment rate by birth cohorts and age groups were shown in Fig. 6. The treatment rates decreased steadily for the two oldest cohorts (1950–1959 and 1940–1949). There was no obvious trend for the 1960–1969 cohort, with the treatment rate varying between 441 and 512 per 100,000 population between 2002 and 2013. This rate increased for the younger cohorts from 2002 but peaked at different times. For the 1970–1979 cohort, the treatment rate increased between 2002 (420 per 100,000 population) and 2007 (591 per 100,000 population), and this increase levelled off after 2007; for the 1980–1989 cohort, this rate increased rapidly between 2002 and 2007 from 213 per 100,000 population to 490 per 100,000 population, and then dropped from 2009 to 2013 (424 per 100,000 population); for the 1990–1999 cohort, the treatment rate increased from 2002 (1.97 per 100,000 population) and peak at 2010 (314 per 100,000 population), and remained stable between 2010 and 2013 (298 per 100,000 population). Analyses by age groups also showed decrease in treatment episodes in all age groups in 2013 (Fig. 7).

## Discussion

This study examined trends in alcohol use over 13 years from 2001 to 2013 in nationally representative samples of the Australian population. The findings suggest a change in trends from increasing to decreasing in alcohol use in Australia. This is consistent with the sales data that shows an increase in per capita consumption between 2001 and 2007, and a decrease thereafter [26]. The decrease in alcohol consumption in the later years were observed in all types of drinkers. There were decreases in prevalence of both high risk drinkers and frequent heavy episodic drinkers. The rate of treatment seeking increased steeply for the younger birth cohort in early 2000 but either stabilized or decreased in later years. In the older cohort, the rate of treatment seeking has decreased steadily since 2002.

These findings are partially consistent with and support Skog’s collectivity theory which suggested that alcohol consumption moves up and down in concert in the population. Collectivity theory is particularly important in explaining long-term consumption trends. Rossow, et al. [7] reported collective changes in alcohol use in Finland, Norway and the US over three decades. This study extends previous research by showing that the timing, rate and degree of changes across different types of drinkers varied during a change of trend. In Australia, while the overall per capita consumption peaked in 2007 [26], the prevalence of abstainers hit the lowest point in 2004 and rebounded thereafter. The consumption per non-high risk drinkers also peaked in 2004, preceding the peak in the whole population. The consumption peak of high risk drinkers occurred in 2010, lagging behind the rest of the population. This differential timing of trend change could explain the divergence in alcohol use and alcohol harm reported in previous studies [10, 11], and this divergence is a transient state during the course of a trend change. At the early stage of a trend change, light drinkers might reduce their consumption or became abstainers while heavy drinkers continue to increase their consumption. Such a difference in the timing of a trend change might be due to barriers between light and heavy drinkers that impede the mutual influence of light and heavy drinkers on each others’ drinking. For example, it is plausible that light and heavy drinkers socialise in very different peer networks, slowing down the mutual influence of light and heavy drinkers. Our findings are consistent with the possibility that the recent change of trend in alcohol use in Australia might have been led by light drinkers, that is, the change in drinking culture started among light drinkers, and has been more slowly propagated to high risk drinkers over the course of a 5 to 6 year period.

Survey data showed signs of a turnaround in alcohol consumption after a decade-long increase, and the treatment data were also consistent with this trend. The overall number of treatment episodes increased from 2002, peaked at 2007 and decreased afterwards. The number of treatment episodes for the two oldest cohorts have been decreasing since 2002. While in the younger cohorts, they have generally increased from 2002 to 2009 but decreased since 2010. The decrease in treatment episodes in recent years was consistent with the decreases in the prevalence of high risk drinkers and heavy episodic drinkers in the same period. However, it should be noted that alcohol remains one of the most significant contributors to disease burden in Australia [11]. In 2010, over 150,000 hospitalisations were directly attributable to alcohol use. Continuous investment in population level prevention and treatment interventions are required to further reduce alcohol related harms. For example, a strict volumetric taxation system on alcohol [27], minimum price per standard drink [28] and raising the legal drinking age to 21 [29] hold potential to further reduce alcohol use and its related harms. As public awareness about the alcohol harm increases, it may become easier to extend the changes in drinking culture to populations that have been more resistant to change [30], such as high risk drinkers in rural areas [31].

### Limitations

Although national data from large, representative samples was used in the current study, it is not without limitations. First, the NDSHSs were based on self-report and under-reporting may occur [14]. There were also changes in data collection methodology, with earlier surveys augmented by face-to-face and/or CATI. Despite these two limitations, a recent study has demonstrated that the NDSHSs were reliable sources of data for monitoring alcohol consumption trends in the Australian population [32]. Second, the NDSHSs excluded individuals without a fixed home and therefore failed to capture some high risk drinkers, such as homeless individuals and those in transient accommodation or institutionalized settings. The use of national treatment data complements these limitations of surveys by providing information on populations with alcohol related problems who may be missed in the household survey. However, the data are confined to patients who nominate alcohol as their primary drug of concern. This may exclude polysubstance dependent users [33]. Third, the ADOTS-NMDS does not include patients of treatment services delivered by private for-profit agencies. However, this is unlikely to affect the results and conclusions in this study, because the private for-profit treatment sector is very small in Australia, unlike the USA. Fourth, the number of treatment episodes could be affected by funding and other factors, such as changes in capacity that would affect the volume of service. Despite these limitations, both of the treatment data and survey data point to the conclusion that alcohol consumption has peaked between 2007 and 2010, and this conclusion is consistent with sales data from the Australia.

## Conclusion

There has been a turnaround in Australian alcohol consumption after a decade-long increase as evidenced by collective decreases in alcohol use across nearly all types of drinkers in recent years. Administrative treatment data also showed a similar trend reversal in treatment seeking, with the treatment rate peaked in 2007/08 and then decreased. The timing of the trend changes differed between different types of drinkers, with light drinker leading the change. Despite the recent decreases in alcohol use, alcohol remained one of the most significant contributors to burden of disease in Australia. Continuous efforts are required to further reduce alcohol related harm in the Australian population.

## Appendix

**Table 1:** Estimates from survey data with 95 % confidence intervals for Figs. 1, 2, 3 and 4

	2001		2004		2007		2010		2013	
	Estimate	(95 % CI)	Estimate	(95 % CI)	Estimate	(95 % CI)	Estimate	(95 % CI)	Estimate	(95 % CI)
Fig. 1 Prevalence of abstainers, high risk drinkers and non-high risk drinkers, 2001–2013										
High risk	18.75	(18.19–19.33)	20.20	(19.62–20.78)	19.83	(19.19–20.48)	19.49	(18.91-20.08)	17.64	(17.05-18.24)
Last year abstinent	16.17	(15.65–16.72)	15.27	(14.77–15.79)	16.77	(16.17–17.39)	19.14	(18.54–19.76)	20.37	(19.72–21.03)
Non-high risk drinker	64.47	(63.78–65.16)	64.17	(63.49–64.85)	63.18	(62.40–63.95)	60.90	(60.16–61.63)	61.21	(60.43–61.98)
Fig. 2 Estimated consumption of high risk drinkers and non-high risk drinkers, 2001–2013										
Mean consumption (High risk drinker; in L)	20.71	(20.23–21.20)	20.79	(20.33–21.25)	21.50	(20.93–22.07)	21.52	(21.02–22.01)	21.03	(20.51–21.55)
Mean consumption (Non-high risk drinker; in L)	2.57	(2.53–2.62)	2.92	(2.87–2.96)	2.88	(2.82–2.93)	2.84	(2.79–2.89)	2.80	(2.74–2.85)
Fig. 3 Prevalence of abstainers, frequent heavy episodic drinkers, and non-frequent heavy episodic drinkers, 2001–2013										
HED 5+ (at least monthly)	27.71	(27.05–28.38)	27.17	(26.54–27.81)	26.96	(26.24–27.69)	26.79	(26.13–27.46)	24.91	(24.23–25.60)
Last year abstinent	16.17	(15.65–16.72)	15.27	(14.77–15.79)	16.77	(16.17–17.39)	19.14	(18.54–19.76)	20.37	(19.72–21.03)
Non heavy episodic drinker (less than monthly)	55.85	(55.12–56.57)	57.55	(56.86–58.25)	56.07	(55.27–56.86)	53.78	(53.04–54.52)	54.15	(53.36–54.93)
Fig. 4 Estimated consumption and number of days engaged in heavy episodic drinking among frequent heavy episodic drinkers, 2001–2013										
Mean consumption (Heavy episodic drinking-Monthly; in L)	15.13	(14.72–15.53)	15.81	(15.41–16.21)	16.44	(15.95–16.94)	16.27	(15.84–16.70)	15.59	(15.14–16.04)
Number of heavy drinking days for heavy episodic drinker (monthly)	92.41	(89.35–95.47)	102.88	(99.99–105.76)	104.87	(101.56–108.19)	100.30	(97.11–103.49)	97.84	(94.38–101.29)

**Table 2:** Population statistics for Figs. 5, 6 and 7

	02/03	03/04	04/05	05/06	06/07	07/08	08/09*	09/10	10/11	11/12	12/13
Fig. 5 Number of treatment episodes per 100,000 population											
Overall	334.83	343.15	338.72	364.33	405.96	437.75	412.85	431.82	430.01	419.16	392.13
Fig. 6 Number of treatment episodes per 100,000 population by birth cohort.											
Birth cohort											
90–99	1.97	10.58	23.72	55.14	87.90	150.30	202.48	263.71	314.54	305.96	298.36
80–89	213.13	257.15	309.52	378.21	443.62	490.32	469.15	490.69	474.76	444.53	424.12
70–79	420.84	439.36	472.25	495.11	560.22	591.82	557.54	592.76	593.84	596.79	570.19
60–69	486.43	496.04	478.50	476.71	512.10	536.75	491.08	500.84	489.33	484.38	441.31
50–59	345.61	339.46	322.83	291.27	307.61	312.59	270.68	261.26	250.28	251.69	221.53
40–49	169.54	158.00	149.81	131.95	134.57	143.49	120.49	123.25	108.98	107.13	88.78
Fig. 7 Number of treatment episodes per 100,000 population by age group.											
Age group											
14–17	110.08	132.28	148.24	197.16	207.26	252.87	259.98	261.40	280.81	248.69	210.55
18–25	298.35	309.67	337.74	387.19	445.28	481.20	452.96	475.55	457.52	390.14	354.46
26–35	449.29	456.38	472.21	487.01	537.50	569.84	534.39	546.24	532.26	504.02	466.07
36–45	472.63	487.03	479.59	488.14	546.86	577.95	556.94	581.99	587.26	597.78	569.46
46–55	309.25	316.70	325.38	314.38	350.53	387.51	354.16	385.49	390.08	415.37	396.34
56–65	146.15	146.96	152.67	148.67	167.62	186.47	170.74	182.49	188.50	197.39	189.30

## Abbreviations

AODT-MNDS: Alcohol and other drug treatment services national minimum dataset
DALY: Disability adjusted life years
NDSHS: National drug strategy household survey
